# PD-L1 expression, *EGFR* and *KRAS* mutations and survival among stage III unresected non-small cell lung cancer patients: a Danish cohort study

**DOI:** 10.1038/s41598-021-96486-2

**Published:** 2021-08-19

**Authors:** Deirdre Cronin-Fenton, Tapashi Dalvi, Naimisha Movva, Lars Pedersen, Hanh Hansen, Jon Fryzek, Elizabeth Hedgeman, Anders Mellemgaard, Torben R. Rasmussen, Norah Shire, Stephen Hamilton-Dutoit, Mette Nørgaard

**Affiliations:** 1 Department of Clinical Epidemiology, Department of Clinical Medicine, Aarhus University & Aarhus University Hospital, Olof Palmes Alle 43-45, 8200 Aarhus N, Denmark; 2 AstraZeneca, Gaithersburg, MD USA; 3 EpidStrategies, Rockville, MD USA; 4 Institute of Pathology, Aarhus University Hospital, Aarhus, Denmark; 5 Department of Oncology, Herlev Hospital, Herlev, Denmark; 6 Danish Lung Cancer Group, Odense, Denmark; 7 Department of Respiratory Medicine, Aarhus University Hospital, Aarhus, Denmark

**Keywords:** Biomarkers, Oncology, Non-small-cell lung cancer

## Abstract

Programmed cell death receptor ligand-1 (PD-L1) expression, KRAS (*KRASm*) and EGFR (*EGFRm*) mutations may influence non-small cell lung cancer (NSCLC) prognosis. We aimed to evaluate PD-L1 expression, *KRASm,* and *EGFRm* and survival among stage III unresected NSCLC patients. Using Danish registries, we collected data on stage III unresected NSCLC patients diagnosed 2001–2012 and paraffin-embedded tumor tissue from pathology archives. We assessed PD-L1 expression in tumors and tumor-infiltrating immune cells (ICs) by immunohistochemistry ($\ge$ 1% threshold for PD-L1+). We genotyped *KRAS* and *EGFR*. Follow-up extended from 120 days post-diagnosis to death, emigration, or 31/12/2014. We computed median survival using Kaplan–Meier methods, and hazard ratios (HRs) using Cox regression associating the biomarkers with death, adjusting for confounders*.* Among 305 patients, 48% had adenocarcinoma; 38% squamous cell carcinoma. Forty-nine percent had PD-L1+ tumors—51% stage IIIA and 26% *KRASm*. Few (2%) patients had *EGFRm.* Median survival in months was 14.7 (95% CI = 11.8–17.9) and 13.4 (95% CI = 9.5–16.3) in PD-L1+ and PD-L1− tumors, respectively. *KRASm* was not associated with death (HR = 1.06, 95% CI = 0.74–1.51 versus wildtype). PD-L1+ tumors yielded a HR = 0.83 (95% CI = 0.63–1.10); PD-L1+ ICs a HR = 0.51 (95% CI = 0.39–0.68). Tumor expression of PD-L1 did not influence survival. PD-L1+ ICs may confer survival benefit in stage III unresected NSCLC patients.

## Introduction

Immunotherapy targets immune checkpoints such as programmed cell death protein-1 (PD-1) and its ligand, PD-L1. Activated T-cells, B-cells and myeloid cells express PD-1 during antigen stimulation^1^. PD-L1 expression on antigen-presenting cells induces T-cell apoptosis by binding PD-1 on T-cells, thereby inhibiting T-cell-mediated antitumor immunity^1,2^. Up to 65% of non-small cell lung cancers (NSCLCs) express PD-L1^3–5^. Yet, the impact of PD-L1 expression on survival is not clear and may depend on other biomarkers^3,6^, including pro-oncogenic mutations, for example involving epidermal growth factor receptor (*EGFR*) or the Kirsten rat sarcoma viral oncogene (*KRAS)*^6,7^. Characterizing the tumor immune microenvironment may help determine which patients could benefit from immunotherapy^8,9^.

The PACIFIC trial, a phase III, placebo-controlled trial, compared durvalumab—a monoclonal antibody that blocks PD-L1 binding to PD-1 and CD80—with observation after chemoradiotherapy in stage III unresected NSCLC patients^10^. Results demonstrated better survival in the treatment arm^10,11^. Yet, the PACIFIC trial did not select patients based on PD-L1 expression. Accordingly, findings may indicate a more general survival benefit of immune checkpoint inhibitor treatments.

Routine clinical care data on the association of PD-L1 expression with *EGFR* and *KRAS* mutations, and survival in stage III unresected NSCLC patients are limited, but may be important to characterize the therapeutic outlook for patients. We therefore conducted a population-based cohort study to investigate the association of PD-L1 expression, mutations in *EGFR* and *KRAS*, with progression-free and overall survival in stage III unresected NSCLC patients in Denmark.

## Methods

All methods used in this study were performed in accordance with guidelines and regulations at Aarhus University. The use of personal data in this study followed the General Data Privacy Regulations and Danish data protection legislation.

### Study cohort

The source population included all men and women aged at least 18 years, resident in Denmark between 2001 and 2012, with follow-up through 2014. The Danish National Health Service provides tax-supported healthcare for the entire population, guaranteeing unfettered access to medical care. Unique civil personal registration (CPR) numbers, assigned to every Danish citizen and resident since 1968, encode gender and date of birth, and enable individual-level electronic record linkage across multiple databases^12,13^.

We ascertained information on all individuals in the source population diagnosed with incident NSCLC (*International Classification of Diseases, Tenth Revision* (ICD-10) diagnosis code of “C34”) between 2001 and 2012, registered in the Danish Lung Cancer Group (DLCG) clinical database, with follow-up through 2014. The DLCG was established in 2000 to register all incident lung cancers diagnosed in Denmark. It has over 95% completeness^14^. Data are registered electronically in the database on completion of a diagnostic procedure or administration of a specific treatment.

We restricted the study cohort to stage III NSCLC patients in the DLCG (Appendix Fig. 1). Using the CPR number, we linked to the Danish National Patient Registry (DNPR) to retrieve information on the receipt of cancer-directed surgery (see Appendix for codes). The DNPR contains data on all nonpsychiatric hospital admissions since 1977 and all outpatient and emergency room hospital contacts since 1995. For each patient contact, a primary diagnosis and any secondary diagnoses are registered according to the ICD codes. The registered information includes diagnostic disease codes, examinations, surgical procedures and certain in-hospital treatments. The validity of the data registered in the DNPR is constantly evaluated^15^. It is high for cancer diagnoses^16,17^, cardiac conditions^18^, acute admissions^19^, as well as cancer-directed treatments^20,21^, among others.

We further restricted the study cohort to unresected stage III patients, which we defined as those who had not undergone cancer-directed surgery within 120 days of their NSCLC diagnosis and staging. This time period of 120 days was chosen to enable removal of patients who underwent neoadjuvant therapy and resection.

We used the CPR number to link to the Danish National Pathology Registry and Pathology Biobank (Patobank) to identify all unresected stage III NSCLC patients with sufficient tumor tissue available for immunohistochemistry (IHC) and molecular analysis. The Pathology Registry was established in 1997 and records data on all pathology examinations conducted in Denmark. In Denmark, all tissue excised during diagnostic histopathological analyses is stored in hospital pathology archives, linked to the Patobank. We collected representative formalin-fixed paraffin-embedded (FFPE) tumor blocks for all patients in the study population with available archived tissue.

This study was approved by the Danish Lung Cancer Group (DLCG) and the Regional Ethics Committee of the Central Denmark Region (Record 1-10-72-14-15). The use of personal data in this study was based on GDPR Art. 9 (2), j), Art. 6 (1), e); and the Danish Data Protection Law §11, and therefore no informed consent is required. We had access to CPR numbers for data linkage purposes. No other personally identifiable information was used.

### PD-L1 expression

For each FFPE tumor block, we cut 5 sections at 4 µm each ensuring a minimum of 100 analysable tumor cells per section. We mounted all sections on Fisher brand SuperFrost Plus positively charged slides (Fisher Scientific, Roskilde, Denmark). We shipped labelled slides at ambient temperature to a certified laboratory contracted by AstraZeneca (Hematogenix; Tinley Park, IL). PD-L1 expression in the tumor cell membrane was assessed by immunohistochemistry (IHC) using the Ventana PD-L1 IHC validated assay (SP263; Ventana Medical Systems, Oro Valley, AZ) and read in batches by manufacturer-trained pathologists in a Clinical Laboratory Improvement Amendments program-certified laboratory (Hematogenix; Tinley Park, IL, USA). A trained pathologist manually evaluated the extent of tumor-infiltrating ICs in hematoxylin & eosin (H&E) stained whole sections.

We considered PD-L1 positivity (PD-L1+) as >  = 1% expression versus PD-L1 negativity (PD-L1−; < 1%)^22^. In sensitivity analyses, we altered the definition of PD-L1 positivity to $\ge$ 25% expression. We further evaluated PD-L1 expression as a continuous variable. In post hoc analyses, we examined the percentage of ICs that expressed PD-L1 and also applied $\ge$ 1% threshold as a cut point. We evaluated the percentage of ICs in the tumor irrespective of PD-L1 expression.

### *KRAS *and *EGFR* genotyping

We cut 5 to 6 sections at 10um each per FFPE tumor block. We extracted DNA from FFPE tissue using standard procedures. We genotyped *KRAS* and *EGFR* using commercial kits (Roche COBAS real-time PCR-based kits)—see Appendix for details of the genotyped mutations. We reported mutation versus wildtype status, in addition to information on invalid test results. We carried out all genotyping in duplicate. We defined *KRAS* and *EGFR* mutations as absent (wildtype), mutated, missing or invalid (the latter when insufficient tissue was available for genotyping). Missing and invalid results for the biomarkers are excluded from the tables.

### Covariates

We ascertained information on tumor size, nodal status, and metastases from the Danish Cancer Registry (DCR), which was established in 1943 and has a completeness of reporting close to 100%^23^. We retrieved information on tumor histology, smoking pack-years, and Eastern Cooperative Oncology Group (ECOG) performance status from the DLCG^24^. From the DNPR, we obtained information on cancer-directed treatments and comorbid diseases recorded up to ten years before NSCLC diagnosis. We used a modified version of the Charlson comorbidity index (CCI) score^25^, removing lung cancer diagnoses. Therefore, patients in our cohort can appear to have a CCI = 0. We categorized the CCI where CCI = 0 denotes no comorbidity; CCI = 1–2 denotes moderate comorbidity; and CCI = 3 + denotes severe comorbidity. We incorporated a variable age of the tumor specimen, which denoted the length of time in days from the date of the tumor specimen to the date of PD-L1 IHC analysis.

### Outcomes

We used the Danish Civil Registration System, which was established in 1968, to ascertain data on all Danish residents including the CPR number and vital status^12^. We defined progression-free survival as the number of days accrued from 120 days after diagnosis (the start of follow-up) to the date of receipt of subsequent cancer-directed therapy (treatment code BWHC) recorded in the DNPR. All-cause mortality was death due to any cause.

### Statistical analysis

We tabulated the study population according to demographic, clinical and treatment characteristics and the biomarkers—*KRAS*, *EGFR*, and PD-L1. In an attempt to avoid immortal time bias due to our definition of unresected disease, follow-up time began 120 days after the date of diagnosis of stage III NSCLC and continued until all-cause mortality according to the Civil Registration System, or the occurrence of disease progression as indicated by treatment codes in the DNPR, or through to the end of the study period (31 December 2014). We censored subjects still alive at the end of follow-up. We used Kaplan–Meier methods to generate survival curves and compute median survival and associated 95% CIs for overall and progression-free survival in the study cohort according to PD-L1 expression, and to *KRAS* or *EGFR* mutation status. We used Cox proportional hazards regression models to compute crude and adjusted hazards ratios (HR) and 95% confidence intervals (95% CI) associating PD-L1 expression in tumor cells, and *KRAS* or *EGFR* mutations, with all-cause mortality and disease progression, adjusting for patient age, gender, age of the tumor specimen, adenocarcinoma versus non-adenocarcinoma histology, and CCI score. We also used Cox models to compute HRs for the association of PD-L1 expression in tumor-infiltrating ICs versus no PD-L1 expression in tumor-infiltrating ICs, with overall and progression-free survival, adjusting HRs for the afore-mentioned covariates. In *post-hoc* analyses, we also used Cox models to compute HRs considering PD-L1 and the extent of ICs as continuous variables with overall and progression-free survival, adjusting HRs for the afore-mentioned covariates. In the case of ties, we used the Breslow method for the log-rank test and the Efron method for Cox regression. All statistical analyses were carried out using SAS version 9.4 (SAS Institute Inc., Cary, NC, USA).

## Results

We included 305 patients with stage III unresected disease (51% stage IIIA and 49% stage IIIB); 55% were aged at least 65 years, and 60% were men. Among stage III unresected patients, 63% were smokers, 2% were non-smokers, and 36% were missing smoking information. All patients in the study cohort received chemotherapy. About one-third of patients received radiation therapy within the first 120 days after diagnosis; 28.5% received radiation therapy after 120 days; and 36.7% did not receive radiation therapy (data not presented). Our study population had similar patient, tumor and treatment characteristics to all stage III unresected patients (data not presented).

**Figure 1:**
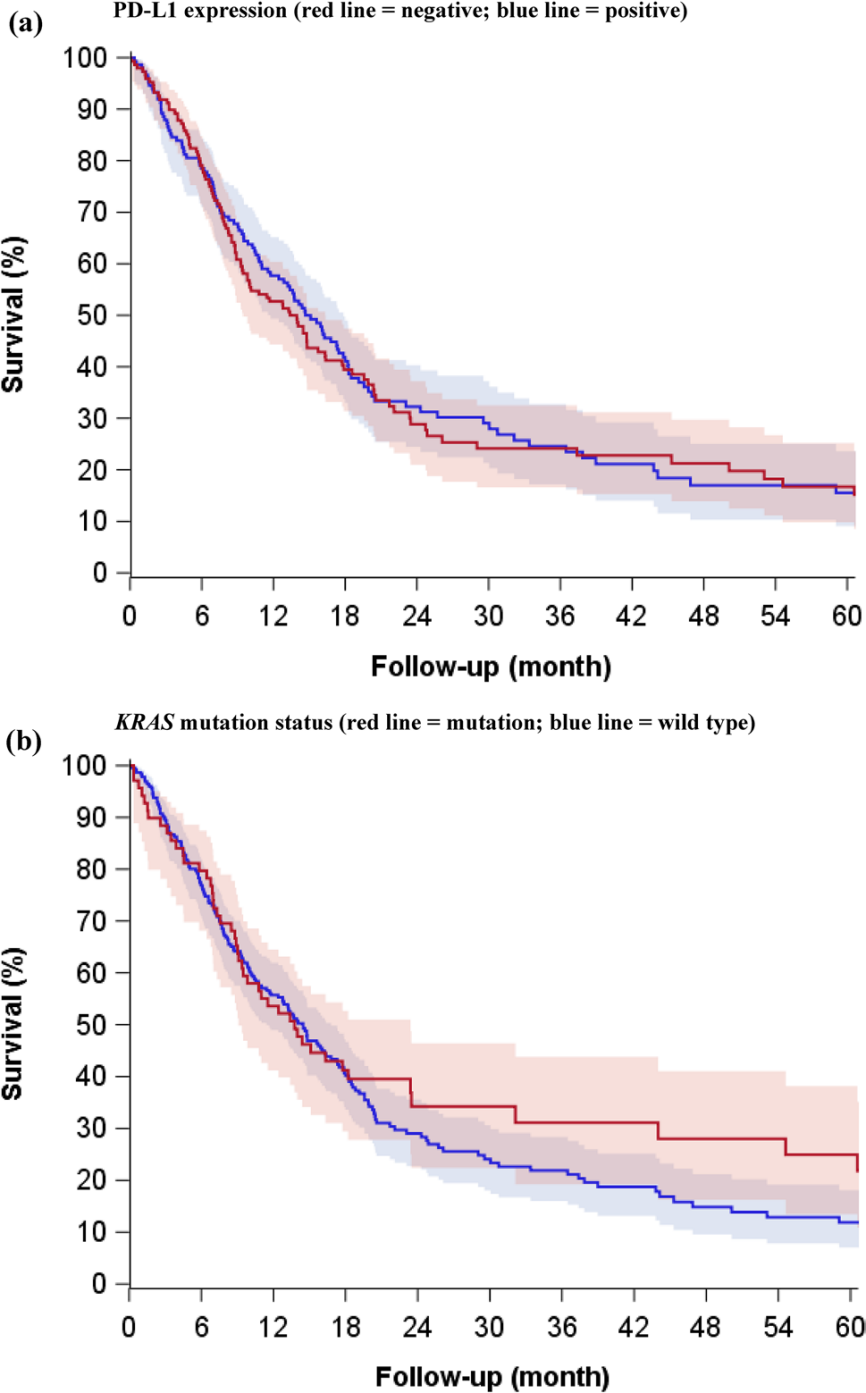
Kaplan–Meier curves illustrating the association of (**a**) PD-L1 expression (negative < 1%; positive >  = 1%), (**b**) *KRAS* mutation status with survival in stage III unresected NSCLC patients diagnosed 2000–2013 with follow-up through 2014, and registered in the Danish Lung Cancer Group Registry. (NSCLC: non-small cell lung cancer; PD-L1: programmed cell death ligand 1; KRAS: Kirsten rat sarcoma virus).

The median age of the tumor specimen was 7.6 years (interquartile range: 3.1–13.0 years). Overall, 49% of patients had PD-L1+ tumors (Table 1). These patients were younger compared with those with PD-L1− tumors (median age 65 versus 67 years, respectively). Adenocarcinoma was the most common histological subtype—44% in PD-L1+ and 52% in PD-L1− tumors. Median overall survival among patients with PD-L1+ and PD-L1− tumors was 14.7 (11.8–17.9) months and 13.4 (9.5–16.3) months, respectively (Fig. 1). The adjusted HR for PD-L1+ versus PD-L1− tumor expression was 0.83 (95% CI = 0.63–1.10), and 1.00 (95% CI = 0.99–1.00) when considering PD-L1 as a continuous variable. The sensitivity analyses where PD-L1+ tumors were those with >  = 25% expression yielded similar findings (HR = 0.80, 95% CI = 0.60–1.07).

**Table 1:** Descriptive characteristics of the cohort of Stage III unresected NSCLC patients diagnosed 2000–2013 and registered in the Danish Lung Cancer Group clinical database, according to tumor PD-L1 expression, and KRAS mutation status*

	Overall^	PD-L1 < 1%	PD-L1 ≥ 1%	*KRAS* Mutation	*KRAS* Wt
	N	N	N	N	N
**Age (years)**					
n	305	148	149	69	226
Mean	–	66.0	64.0	62.0	66.0
SD	–	8.0	9.0	10.0	8.0
Median	–	67.0	65.0	63.0	66.0
Min	–	39.0	38.0	38.0	41.0
Max	–	82.0	82.0	79.0	82.0
Missing	–	0.0	0.0	0.0	0.0
**Age group (years)**	**N (%)**	**N (%)**	**N (%)**	**N (%)**	**N (%)**
18–59	77 (25.2)	29 (19.6)	47 (31.5)	28 (40.6)	48 (21.2)
60–64	61 (20.0)	28 (18.9)	31 (20.8)	10 (14.5)	48 (21.2)
65–69	77 (25.2)	40 (27.0)	33 (22.1)	15 (21.7)	60 (26.5)
70 -	90 (29.5)	51 (34.5)	38 (25.5)	16 (23.2)	70 (31.0)
**Sex**					
Female	122 (40.0)	63 (42.6)	54 (36.2)	44 (63.8)	76 (33.6)
Male	183 (60.0)	85 (57.4)	95 (63.8)	25 (36.2)	150 (66.4)
**Vital status**					
Alive	75 (24.6)	37 (25.0)	3724.8)	21 (30.4)	52 (23.0)
Dead	230 (75.4)	111 (75.0)	112 (75.2)	48 (69.6)	174 (77.0)
**Smoking status**					
Non-smoker	5 (1.6)	4 (2.7)	0 (0.0)	0 (0.0)	5 (2.2)
Smoker	191 (62.6)	93 (62.8)	93 (62.0)	44 (63.8)	139 (61.5)
Missing	109 (35.7)	51 (34.5)	55 (36.0)	25 (36.2)	82 (36.3)
**Charlson comorbidity index (CCI) score**					
CCI = 0	150 (49.2)	70 (47.3)	76 (51.0)	32 (46.4)	115 (50.9)
CCI = 1–2	74 (24.3)	38 (25.7)	35 (23.5)	15 (21.7)	55 (24.3)
CCI = 3 +	81 (26.6)	40 (27.0)	38 (25.5)	22 (31.9)	56 (24.8)
**ECOG performance status**					
(0) Fully active, no restrictions	101 (33.1)	50 (33.8)	48 (32.2)	25 (36.2)	72 (31.9)
(1) Limited in physically demanding activities	61 (20.0)	33 (22.3)	28 (18.8)	13 (18.8)	45 (19.9)
(2) In bed ≤ 50% of the time	24 (7.9)	9 (6.1)	15 (10.1)	6 (8.7)	18 (8)
(3) In bed > 50% of the time/completely disabled/dead	4 (1.3)	3 (2.1)	1 (0.7)	0	4 (1.7)
Missing	110 (36.1)	50 (33.8)	56 (37.6)	23 (33.3)	84 (37.2)
Unknown	5 (1.6)	3 (2.0)	1 (0.7)	2 (2.9)	3 (1.3)
**Primary tumor location**					
Right Lung	106 (34.8)	51 (34.5)	53 (35.6)	23 (33.3)	77 (34.1)
Left Lung	83 (27.2)	44 (29.7)	37 (24.8)	22 (31.9)	60 (26.5)
Bilateral	5 (1.6)	2 (1.4)	3 (2.0)	1 (1.4)	4 (1.8)
Missing	111 (36.4)	51 (34.5)	56 (37.6)	23 (33.3)	85 (37.6)
**Histology type**					
Squamous or epidermoid	117 (38.4)	56 (37.5)	59 (39.6)	10 (14.5)	102 (45.1)
Adenocarcinoma	148 (48.5)	77 (52)	66 (44.3)	55 (79.7)	90 (39.8)
Large cell carcinoma	5 (1.6)	0 (0)	5 (3.4)	0 (0.0)	5 (2.2)
Adenosquamous carcinoma	5 (1.6)	1 (0.7)	3 (2.0)	2 (2.9)	3 (1.3)
Carcinoids	3 (1.0)	2 (1.4)	1 (0.7)	0 (0.0)	3 (1.3)
Non-small cell carcinoma	27 (8.9)	12 (8.1)	15 (10.1)	2 (2.9)	23 (10.2)

*NSCLC: non-small cell lung cancer; PD-L1: programmed cell death ligand 1; KRAS: Kirsten rat sarcoma virus.

^In the cohort of 305 stage III unresected patients, 9 had a missing/invalid PD-L1 result; 10 had a missing KRAS result.

Only 2% (n = 6 patients) of the study population had tumors that harbored *EGFR* mutations. This low number precluded a more extensive evaluation of the association of *EGFR* mutations with survival in our study.

Twenty-three percent of patients had *KRAS* mutated tumors. Median age at diagnosis was 63 years versus 66 years for patients with mutated versus wildtype tumors. Patients with *KRAS* mutated tumors were more often female (64% versus 34%), and had lower median smoking pack-years (30 versus 40 pack-years). Adenocarcinoma histology was more frequent in patients with *KRAS* mutated tumors (80%). The frequency of adenocarcinomas (40%) and squamous cell carcinomas (45%) was similar in *KRAS* wildtype tumors. Median overall survival of patients with *KRAS* mutated and wildtype tumors was 13.8 (9.1–23.4) months and 14.5 (11.4–16.8) months, respectively (Fig. 1), with a corresponding adjusted HR of 1.06 (95% CI = 0.74–1.51). PD-L1+ tumors were more frequent among *KRAS* mutated than wildtype cancers (26% versus 18%, respectively) (data not presented).

The analyses investigating the association of PD-L1 and *KRAS* mutations with disease progression were similar to those of mortality (Table 2).

**Table 2:** Hazard ratio (HR) and associated 95% confidence interval (95% CI) of mortality and progression (defined as time to next treatment) according to PD-L1 expression, and *KRAS* mutation status among stage III unresected NSCLC patients diagnosed 2000–2013 and registered in the Danish Lung Cancer Group clinical database^

Model	Adjusted HR* Mortality (95% CI)	Adjusted HR* Progression (95% CI)
PD-L1% tumor cell membrane staining (**≥ **1% versus < 1%)	0.83 (0.63–1.10)	0.78 (0.58–1.05)
PD-L1 (continuous)	1.00 (0.99–1.00)	1.00 (0.99–1.00)
KRASm (vs wildtype)	1.06 (0.74–1.51)	1.06 (0.74–1.50)
PD-L1+ ICs (**≥ **1% versus < 1%)	0.51 (0.39–0.68)	0.50 (0.38–0.66)
% ICs§ (continuous)	0.96 (0.93–0.99)	0.96 (0.93–0.99)

*Adjusted for age, sex, histology (adenocarcinoma versus other), Charlson comorbidity index score and age of the tumor specimen.

^§^% ICs: % tumor infiltrating immune cells.

^NSCLC: non-small cell lung cancer; PD-L1: programmed cell death ligand 1; KRAS: Kirsten rat sarcoma virus.

### Post hoc analyses

In total, 109 (36%) patients had tumors with PD-L1− ICs; 187 (61%) patients had tumors with PD-L1+ ICs (Appendix Table 1). Adenocarcinoma was the most common histological subtype—49% and 48% among tumors with PD-L1+ and PD-L1− ICs, respectively. The proportion of patients who had died by the end of follow-up was 94% for those with PD-L1− ICs and 70% of those with PD-L1+ ICs. Median overall survival among patients with tumors bearing PD-L1− and PD-L1+ ICs was 8.7 (7.0–10.4) months and 17.9 (14.8–19.9) months, respectively. PD-L1 expression in ICs was associated with a survival benefit (adjusted HR = 0.51, 95% CI = 0.39–0.68). The presence of ICs in the tumor sample (considered as a continuous variable) was associated with a survival benefit (adjusted HR = 0.96, 95% CI = 0.93–0.99), irrespective of PD-L1 status.

## Discussion

We found that PD-L1 expression in stage III unresected NSCLC tumors was not associated with patient survival or disease progression. Yet, PD-L1 expression in ICs and the extent of ICs in the tumor were associated with survival benefit. Few patients in our study had tumors with *EGFR* mutations. *KRAS* mutation status was associated with PD-L1 expression in tumors, but not with patient survival.

Several issues warrant consideration when interpreting our findings. The diagnostic period of our cohort preceded the use of PD-L1 immune checkpoint inhibitors in routine clinical care in Denmark. Our study therefore examines the potential prognostic rather than predictive role of these biomarkers. The use of Danish healthcare registries facilitated a nationally representative sample of stage III unresected NSCLC patients with complete follow-up. Use of the DLCG ensured high quality data on NSCLC diagnosis and treatment. Individual-level linkage via the CPR number across the Danish registries provided information on several potential confounders including comorbidity, smoking pack-years, and ECOG performance status, thereby eliminating recall bias. Linkage to the Danish Pathology Registry and Patobank facilitated efficient retrieval of NSCLC tumor tissues archived at the time of diagnosis from all patients with available material. Nonetheless, it is important to note, that the pathological diagnosis of NSCLC in Denmark is often based on cytological evaluation only^26^. The Ventana PD-L1 assay is not designed to assay PD-L1 expression in cytology specimens. Therefore, while all patients included in our study were inoperable at diagnosis, they needed to have sufficient quantities of histological tumor tissue available in the pathology archives for PD-L1 testing. This tissue was from either biopsies or surgical resections—where surgery occurred more than 120 days after initial NSCLC diagnosis. Accordingly, our study cohort may include a selected group of stage III unresected NSCLC patients. In addition, PD-L1 status in our study may not necessarily represent actual PD-L1 status in the tumors at the time of diagnosis, in those cases in which tumor specimens were from surgical resections performed more than 120 days after diagnosis. In these cases the tissue may not be treatment naïve, and PD-L1 expression, which can change in response to chemotherapy in NSCLC^27^, may not reflect expression at the time of diagnosis. We did not incorporate information on administered therapies. PD-L1 expression may deplete over time particularly in tumor specimens that are more than three years old^28^. We therefore adjusted for the age of the tumor specimen to account for the elapsed time from biopsy/surgery to PD-L1 testing. Both smoking and ECOG performance status influence NSCLC survival. Unfortunately, we had quite a high proportion of missing information on smoking status and ECOG performance status. We were therefore unable to incorporate these data into our analyses.

NSCLC is an intrinsically heterogeneous disease with documented discordance of PD-L1 expression across different tumor areas and immune cells^29^. Some studies document higher PD-L1 expression in immune cells compared with tumor cells, even in the same tumor region^30,31^. No consensus exists regarding the threshold for PD-L1 expression, or the exact tumor area in which to assay it. In some cancers—for example Hodgkin lymphoma—PD-L1 gene amplification predicts response to anti-PD-1 therapy^32,33^. However, PD-L1 gene copy number variation has not been shown to play a role in NSCLC and was, therefore, not investigated in our study.

The prognostic value of PD-L1 expression in stage III unresected NSCLC remains unclear. Our observed null association agrees with some, but not all^34–36^ of the published literature. Meng et al. observed poorer prognosis associated with $\ge$ 10% PD-L1 expression, hypothesizing that this could be attributable to differences in the tumor immune microenvironment in squamous versus non-squamous NSCLC^37^. Tokito et al. also reported no association of PD-L1 ($\ge$ 5%) with survival^38^. Their study was small including only 74 patients with stage III NSCLC, all of whom were treated with chemoradiotherapy. In a cohort of 117 stage III unresected patients, Vrankar and colleagues also observed no evidence of an association of PD-L1 expression with survival^39^. However, in an earlier study, Vrankar and colleagues documented poorer survival associated with PD-L1 expression (> = 5%) in 102 stage III unresectable patients treated with chemoradiotherapy^36^. In both of their studies, over 50% of the included patients had squamous cell carcinoma contrasting with our predominantly adenocarcinoma cohort. Higher PD-L1 expression has been associated with squamous cell carcinoma compared with other histological subtypes^40^, and lower survival has been documented in adenocarcinoma patients with high ($\ge$ 50% PD-L1) compared with lower expression. Studies have also reported higher PD-L1 expression with advancing disease stage^41,42^; which may have been difficult to detect in our relatively uniform unresected stage III population. A study of advanced NSCLC showed the dynamic PD-L1 expression as tumors progressed from primary to metastatic NSCLC^43^. Taken together, reasons underlying the apparently conflicting findings on the role of PD-L1 expression in prognosis of stage III unresectable NSCLC may include differences in the antibodies used, variable threshold levels for PD-L1, and interstudy variation in terms of the histological subtypes. Studies of stage III unresected NSCLC have been small ranging in size from 31 patients^35^ to our study at 305 patients, and so estimates are imprecise.

A meta-analysis by Li and colleagues showed that the association of PD-L1 with survival varied depending on the extent of PD-L1 expression in tumors^44^. They incorporated 50 studies ranging in size from 36 to 1070 patients, with any stage NSCLC. Although they observed little evidence of an association at > 50% expression, they did note an association at > 1% and > 5% thresholds. Our findings remained robust in sensitivity analyses where we modified the threshold for PD-L1 expression to $\ge$ 25%. Most studies included in the meta-analysis stemmed from Asian populations and indicated an association of PD-L1 expression with higher mortality. However, studies in non-Asian populations (n = 11) observed little evidence of an association between tumor PD-L1 expression and overall survival in NSCLC patients. Two out of the fifty studies included in the meta-analysis utilized the SP263 antibody—Igawa et al. observed no association of PD-L1 with survival in stage I-III patients^45^, whereas Wu et al. documented lower survival associated with PD-L1 expression in a cohort of stage I-IV NSCLC patients^46^, though estimates were imprecise. The meta-analysis by Li and colleagues noted that PD-L1 expression detected in surgical specimens was associated with survival but expression in biopsy samples was not^44^. This may reflect the afore-mentioned heterogeneity of PD-L1 expression in tumors, and may have influenced our findings in our cohort of unresected stage III patients.

Despite the lack of association of *tumor* expression of PD-L1 and survival, we found lower mortality risk among patients with tumors carrying increased numbers of ICs, and among those with PD-L1 positive ICs. Tumor-infiltrating ICs, and specifically tumor-infiltrating lymphocytes have been associated with better prognosis in a study of 197 Chinese patients with stage I-III NSCLC^37^. Among stage III patients, Gettinger and colleagues observed survival benefit associated with tumor-infiltrating lymphocytes, in addition to a survival benefit upon treatment with immune checkpoint blockers, irrespective of tumor mutational load and PD-1 expression^47^. It is also noteworthy that the PACIFIC trials observed lower lung cancer progression among patients treated with durvalumab irrespective of PD-L1 expression. It would be interesting to see if the proportion of PD-L1+ ICs in the tumor microenvironment could predict response to treatment with immune checkpoint inhibitors^48^.

Few patients in our study had *EGFR* mutations, so we were unable to consider their association with PD-L1 expression, *KRAS* mutations*,* or survival. The prevalence of NSCLCs with *KRAS* mutations in our study compares to published studies^7, 49^. Our findings are consistent with a meta-analysis published in 2018 including 26 studies and 7541 patients, which suggested that PD-L1 expression was higher in tumors with *KRAS* mutations compared with wildtype^50^. The lack of association of *KRAS* mutations with survival in our population of stage III unresected NSCLC patients agrees with some^51^, but not all^52–54^ published studies. A meta-analysis from 2005 including 3620 NSCLC patients of all stages at diagnosis suggested that *KRAS* mutations were associated with poor prognosis^55^. *KRAS* mutations may modify the response to *EGFR*-inhibitors in NSCLC^56^, or to immune checkpoint inhibitor therapy^57^. Unfortunately, we were unable to evaluate their predictive utility to exact treatment regimens as such specific medication data were not systematically available in the DNPR for the diagnostic period of our study.

Findings from our study can be used as a benchmark when evaluating the prognostic ability of PD-L1 expression (and *KRASm*) in stage III unresected NSCLC patients. However, the diagnostic period of our study cohort preceded the widespread dissemination of immune checkpoint inhibitors in routine clinical practice. We therefore could not evaluate the predictive ability of these biomarkers on the effectiveness of PD-1/PD-L1 immune checkpoint inhibitor therapies.

In conclusion, findings from this Danish cohort study suggest that PD-L1 expression in tumors was not associated with survival in NSCLC patients. However, the presence of ICs and PD-L1 expression in ICs appeared to be associated with a survival benefit in unresected stage III NSCLC patients.

### Ethics approval and consent to participate

This study was approved by the Danish Lung Cancer Group (DLCG) and the Regional Ethics Committee of the Central Denmark Region (Record 1-10-72-14-15). The use of personal data for the project has been approved by Aarhus University in accordance with General Data Privacy Regulations and the Danish data protection legislation and does not require informed consent from the research subjects or approval from governmental or ethical bodies.

### Consent for publication

This project is based on registry data and under Danish law does not require informed consent.

## Supplementary Information

Supplementary Information.

## Data Availability

Under Danish law, the datasets used in the current study are not permitted to be accessed publicly.
