# Supplementary material for: PD-L1 expression, EGFR and KRAS mutations and survival among stage III unresected non-small cell lung cancer patients: a Danish cohort study
Source: Sci Rep. 2021 Aug 19;11:16892. doi: 10.1038/s41598-021-96486-2 (PMC8377072; doi:10.1038/s41598-021-96486-2)
Supplement: Supplementary file 1 — Supplementary Information. [file 41598_2021_96486_MOESM1_ESM.docx]

**APPENDIX**

*Lung cancer surgery codes:*

KGD codes in the DNPR excluding KGDB, KGDC, KGDD.

*Genotyped mutations:*

We assayed the following mutations in *EGFR*: Exon 18: G719X; Exon 19: Exon 19 deletion; Exon20: T790M, S768I, Exon 20 Insertion; and Exon 21: L858R. We assayed the following mutations in *KRAS*: Exon 2: Codon 12 or codon 13 mutations; Exon 3: Codon 61 mutations.

**Appendix Figure 1:** Flow chart showing eligible and excluded formalin fixed paraffin-embedded tumor (FFPET) tissue blocks available from the Danish National Pathology Registry. Stage III unresected patients diagnosed in Denmark from 2001 through 2012.


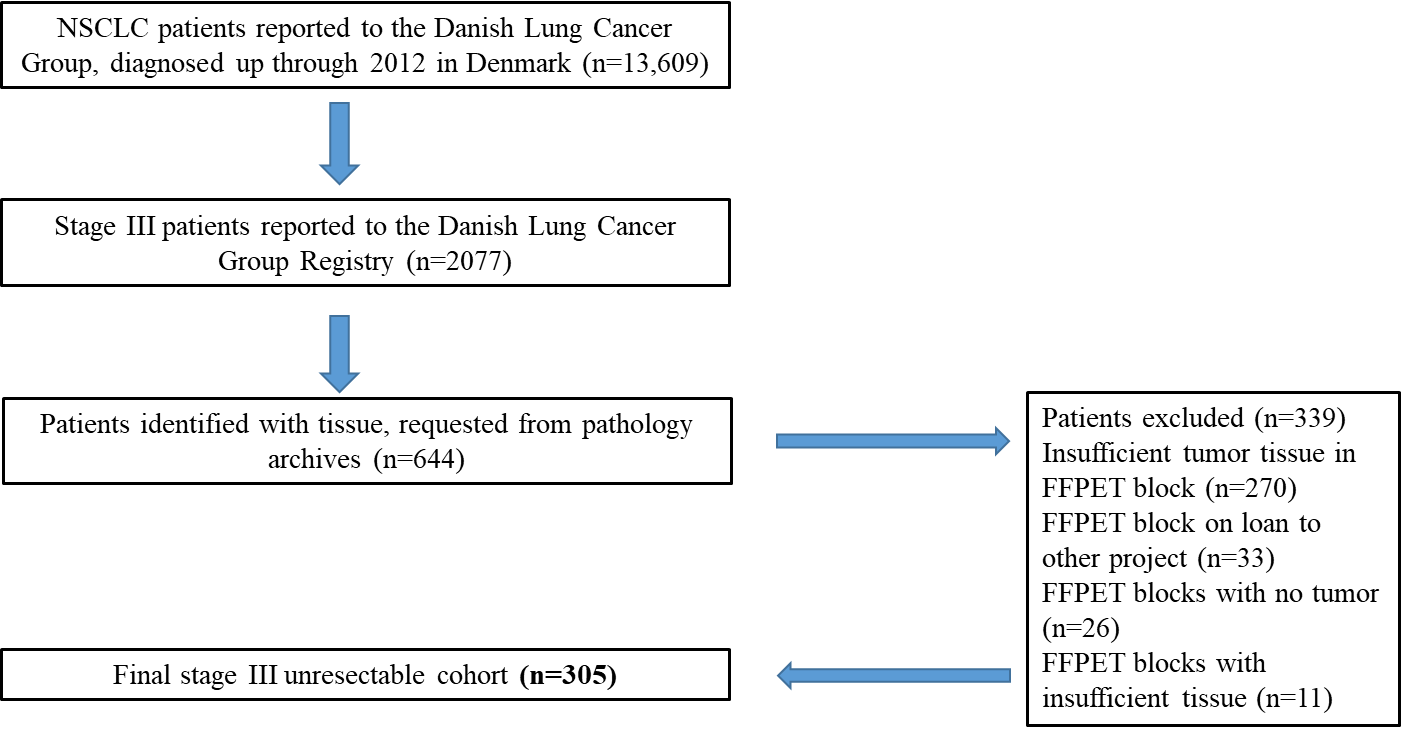


**Appendix Table 1: Descriptive characteristics of the cohort of Stage III unresected NSCLC patients diagnosed 2000-2013 and registered in the Danish Lung Cancer Group clinical database, according to PD-L1 expression in tumor infiltrating lymphocytes (ICs)**

|  | **PD-L1<1% ICs** | **PD-L1>1% ICs** |
| --- | --- | --- |
| **Age group (years)** | **N (%)** | **N (%)** |
| 18 - 59 | 28 (25.7) | 47 (25.1) |
| 60 - 64 | 23 (21.1) | 36 (19.3) |
| 65 - 69 | 27 (24.8) | 46 (24.6) |
| 70 - | 31 (28.4) | 58 (31.0) |
| **Sex** |  |  |
| Female | 47 (43.1) | 69 (36.9) |
| Male | 62 (56.9) | 118 (63.1) |
| **Vital Status** |  |  |
| Alive | 10 (9.2) | 63 (33.7) |
| Dead | 99 (90.8) | 124 (66.3) |
|  |  |  |
| **Smoking status** |  |  |
| Non-smoker | 1 (0.9) | 4 (2.1) |
| Smoker | 71 (65.1) | 115 (61.5) |
| Missing | 37 (33.9) | 68 (36.4) |
|  |  |  |
| **Histology type** |  |  |
| Squamous cell | 40 (36.7) | 75 (40.1) |
| Adenocarcinoma | 58 (53.2) | 84 (44.9) |
| Large cell carcinoma | 0 (0) | 5 (2.7) |
| Adenosquamous carcinoma | 1 (0.9) | 3 (1.6) |
| Carcinoids | 1 (0.9) | 2 (1.1) |
| Non-small cell carcinoma | 9 (8.3) | 18 (9.6) |
|  |  |  |
